# Candidate genes for alcohol preference identified by expression profiling in alcohol-preferring and -nonpreferring reciprocal congenic rats

**DOI:** 10.1186/gb-2010-11-2-r11

**Published:** 2010-02-03

**Authors:** Tiebing Liang, Mark W Kimpel, Jeanette N McClintick, Ashley R Skillman, Kevin McCall, Howard J Edenberg, Lucinda G Carr

**Affiliations:** 1 Indiana University School of Medicine, Department of Medicine, IB424G, 975 West Walnut Street, Indianapolis, IN 46202, USA; 2 Indiana University School of Medicine, Department of Psychiatry, PR116, Indianapolis, IN 46202, USA; 3 Indiana University School of Medicine, Department of Biochemistry and Molecular Biology, 635 Barnhill Dr., Indianapolis, IN 46202, USA; 4 Washington University Orthopedics, Campus Box 8233, One Children's Place, Suite 4S60, St Louis, Missouri 63110, USA

## Abstract

**Background:**

Selectively bred alcohol-preferring (P) and alcohol-nonpreferring (NP) rats differ greatly in alcohol preference, in part due to a highly significant quantitative trait locus (QTL) on chromosome 4. Alcohol consumption scores of reciprocal chromosome 4 congenic strains NP.P and P.NP correlated with the introgressed interval. The goal of this study was to identify candidate genes that may influence alcohol consumption by comparing gene expression in five brain regions of alcohol-naïve inbred alcohol-preferring and P.NP congenic rats: amygdala, nucleus accumbens, hippocampus, caudate putamen, and frontal cortex.

**Results:**

Within the QTL region, 104 *cis*-regulated probe sets were differentially expressed in more than one region, and an additional 53 were differentially expressed in a single region. Fewer *trans*-regulated probe sets were detected, and most differed in only one region. Analysis of the average expression values across the 5 brain regions yielded 141 differentially expressed *cis*-regulated probe sets and 206 *trans*-regulated probe sets. Comparing the present results from inbred alcohol-preferring *vs*. congenic P.NP rats to earlier results from the reciprocal congenic NP.P *vs*. inbred alcohol-nonpreferring rats demonstrated that 74 *cis*-regulated probe sets were differentially expressed in the same direction and with a consistent magnitude of difference in at least one brain region.

**Conclusions:**

*Cis*-regulated candidate genes for alcohol consumption that lie within the chromosome 4 QTL were identified and confirmed by consistent results in two independent experiments with reciprocal congenic rats. These genes are strong candidates for affecting alcohol preference in the inbred alcohol-preferring and inbred alcohol-nonpreferring rats.

## Abstract 2

Transcriptional profiling of specific regions of inbred rat brains reveals genes associated with alcohol preference in a known QTL locus on chromosome 4

## Background

Alcoholism has a substantial genetic component, with estimates of heritability ranging from 50 to 60% for both men and women [1-3]. The associations of several genes with risk for alcoholism have been replicated in human studies: *GABRA2 *[4-11], *ADH4 *[12-14], and *CHRM2 *[15,16]. Several other genes have been associated with alcoholism or related traits and await replication [17,18], including *TAS2R16 *[19,20], *NTRK2 *[21], *GABRG3 *[22], *GABRA1 *[23], *OPRK1 *and *PDYN *[24,25], *NFKB1 *[26], *ANKK1 *[27], *ACN9 *[28], *TACR3 *[29], *CHRNA5 *[30], *SNCA *[31], *NPY *[32,33], and *NPY *receptors [34].

Selected strains of rodents that differ in voluntary alcohol consumption have been valuable tools to aid in dissecting the genetic components of alcoholism [35-38]. The alcohol-preferring (P) and -nonpreferring (NP) rat lines were developed through bi-directional selective breeding from a randomly bred, closed colony of Wistar rats on the basis of alcohol preference in a two-bottle choice paradigm [36]. P rats display the phenotypic characteristics considered necessary for an animal model of alcoholism [39,40]. Subsequently, inbred alcohol-preferring (iP) and -nonpreferring (iNP) strains were established; these inbred strains maintain highly divergent alcohol consumption scores [41]. Due to the physiological and genetic similarity between humans and rats, iP and iNP rats can be studied to identify important genetic factors that might influence predisposition to alcoholism in humans.

A highly significant quantitative trait locus (QTL) that influenced alcohol preference was identified on chromosome 4, with a maximum LOD score of 9.2 in a cross between iP and iNP rats [41]. The chromosome 4 QTL acts in an additive fashion and accounts for approximately 11% of the phenotypic variability. This approximately 100 million bases (Mb) QTL region is likely to harbor genes that directly contribute to alcohol preference. Several candidate genes identified in human studies *(SNCA*, *NPY*, *CHRM2*, *TAS2R16*, and *ACN9*) have homologs located within this rat chromosome 4 QTL. *Snca *and *Npy *have been shown to be differentially expressed between these two strains [42,43].

**Figure 1:**
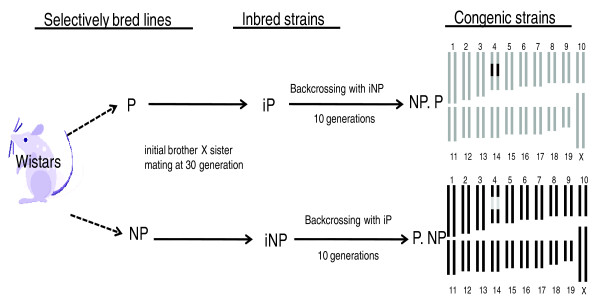
**Development of reciprocal congenic strains**. Alcohol-preferring (P) and alcohol-nonpreferring (NP) rats were selectively bred for high and low alcohol drinking from a closed colony of Wistar rats [36]. Inbreeding was initiated at generation 30 to create the inbred P (iP) and iNP rats [41]. Chromosome 4 reciprocal congenic rats were developed in which the iP chromosome 4 QTL interval from *D4Rat119 *to *D4Rat55 *was transferred to the iNP (NP.P-(*D4Rat119-D4Rat55*)) and the iNP chromosome 4 QTL interval was transferred to the iP (P.NP-(*D4Rat119-D4Rat55*)) [44]. Genotyping of *D4Rat15*, *D4Rat119*, *D4Rat55*, and D4Rat *192 *revealed that the recombination location was between *D4Rat15 *and *D4Rat119 *and between *D4Rat55 *and *D4Rat192 *[44].

Reciprocal congenic strains (Figure 1) in which the iP chromosome 4 QTL interval was transferred to the iNP (NP.P-(*D4Rat119*-*D4Rat55*) and the iNP chromosome 4 QTL interval was transferred to the iP (P.NP-(*D4Rat119*-*D4Rat55*) exhibited the expected effect on alcohol consumption: that is, the consumption correlated with the strain that donated the chromosome 4 QTL interval [44]. (In this paper, the reciprocal congenic strains will be referred to as NP.P and P.NP.) Thus, the chromosome 4 QTL region is, in part, responsible for the disparate alcohol consumption observed between the iP and iNP rats.

Identifying the genes in the chromosome 4 interval that underlie the phenotype has been difficult. We adopted a strategy of using transcriptome analysis to determine which genes are altered in expression in the congenic strains; this is a powerful approach toward gene identification [45-47]. Using this approach reduces the 'noise' from unrelated differences in gene expression, because the two strains are identical except for the QTL sequences, and thereby increases the specificity with which genes contributing to the specific phenotype can be detected.

Previous transcriptome profiling of the NP.P congenic strain and the iNP background strain identified 35 candidate genes in the chromosome 4 QTL that were *cis*-regulated in at least one of the five brain regions studied [47]. Nucleus accumbens, frontal cortex, amygdala, hippocampus, and caudate putamen were examined, based on their inclusion in the mesolimbic and mesocortical systems, both of which are important in the initiation and maintenance of goal-directed and reward-mediated behaviors [48,49]. In the present paper, we compare the iP background strain with the reciprocal congenic strain (P.NP) to identify *cis *and *trans *differentially expressed genes. The strategy of identifying differentially expressed genes in congenic strains and using comparisons between the reciprocal congenic strains to further support the differences allowed us to identify genes that are strong candidates for affecting alcohol preference.

## Results

### *Cis*-regulated genes

Because alcohol preference in the congenic strains correlated with the strain origin of the introgressed region, our primary hypothesis was that the genes in that region contributing to the phenotype would differ in expression as a result of *cis*-acting elements. Transcriptome analyses were performed to detect differences in gene expression between iP and congenic P.NP rats in five brain regions: nucleus accumbens, frontal cortex, amygdala, hippocampus, and caudate putamen.

**Figure 2:**
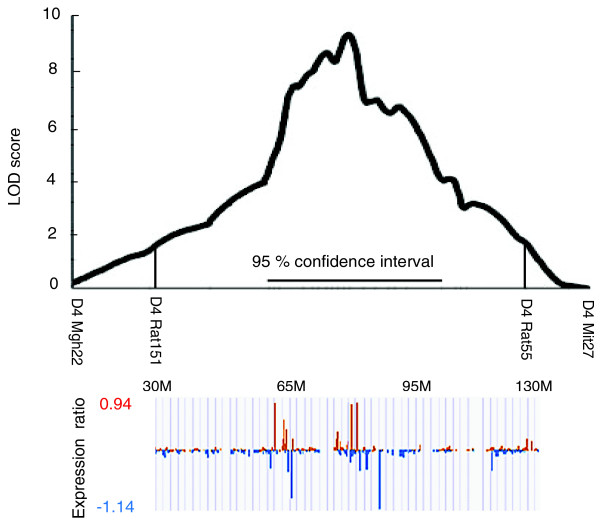
**Differentially expressed probe sets within the chromosome 4 QTL interval**. Top panel: chromosome 4 QTL lod plot, based on reanalysis of our original data from [101] plus additional genotyping, using the current positions of the markers. The 95% confidence interval for the QTL is indicated by a horizontal line. The transferred region of the QTL is indicated by vertical lines. Bottom panel: The expression (E) ratios (E_P.NP_-E_iP_)/E_iP _of the probe sets from approximately 30 Mb to 130 Mb were aligned with the lod plot in the top panel.

Of the probe sets differentially expressed in the introgressed region of chromosome 4, many are located within the 95% confidence interval of the QTL (54.8 to 105 Mb). (Figure 2) The number of differentially expressed probe sets (false discovery rate (FDR) ≤ 0.25) within the QTL was similar in each of the 5 brain regions, ranging from 72 in the nucleus accumbens to 89 in the hippocampus (Table 1). most probe sets significant in any one brain region were significant in multiple regions; 104 of the 157 *cis*-regulated probe sets showed differential expression in more than one brain region. Only 8 to 21% of those detected in any single region were detected in only that region (Table 1). Analysis of the average level of gene expression across all 5 regions showed 141 probe sets that significantly differed between the strains; this included 19 probe sets not detected in any of the individual regions (Table 1; also see Table S1 in Additional file 1, which includes a list of significant differentially expressed *cis*-regulated genes).

**Table 1:** Number of differentially expressed probe sets in the iP vs P.NP Comparison

	Nucleus accumbens	Amygdala	Frontal cortex	Hippo-campus	Caudate putamen	At least one brain region	Multiple brain regions	Combined regions
Significant *cis*-regulated probe sets								
Total	72	74	78	89	82	157	104	141
Single brain region only	11	8	7	10	17			
Only significant in combined								19
								
Significant *trans*-regulated probe sets								
Total	14	7	16	17	54	85	10	206
Single brain region only	9	2	8	10	46			
Only significant in combined								143

*Cis*-regulated probe sets are those located in the chromosome 4 QTL interval; *trans*-regulated probe sets are located in the remainder of the genome. The first five columns show the number of *cis*- and *trans*-regulated probe sets that differ between iP and P.NP in each individual brain region. 'At least one brain region' shows the total number of unique probe sets that differed in one or more regions. 'Multiple brain regions' shows the total number of unique probe sets that differed in at least two of the five brain regions. 'Average expression' shows probe sets that differ when the average expression across the five regions in each animal was analyzed. 'Single brain region only' shows the number of unique probe sets significant in only that brain region. 'In average only' shows unique probe sets that were significant only in analysis of the average level of expression across the five regions in each animal.

### *Trans*-regulated genes

To detect *trans*-regulated genes (genes identical in the two strains that are differentially expressed due to variations in a regulatory gene located within the chromosome 4 region), the remainder of the genome (everything except the chromosome 4 QTL region) was analyzed. Differentially expressed genes are not concentrated on any chromosome, other than chromosome 4 (Table S2 in Additional file 1). Although the total number of genome probe sets analyzed was much greater than the QTL probe sets (for example, 23,050 probe sets were used in the averaged analysis, versus 960 in the *cis*-analysis above; see Materials and methods for details), fewer *trans*-regulated probe sets were differentially expressed in each region or in multiple regions (Table 1). Unexpectedly, we found 54 significant probe sets in the caudate putamen, of which 46 were only significant in that brain region. The analysis of the average level of gene expression across all 5 regions was more powerful than the analyses of individual brain regions; 206 *trans*-regulated probe sets differed, including 143 that did not differ in any individual region (Table 1; also see Table S2 in Additional file 1, which includes a list of differentially expressed *trans*-regulated genes).

Some of the *trans*-regulated genes were previously implicated in drug or alcohol addiction, including *Pnlip *(pancreatic lipase) [50], *Homer1 *(homer homolog 1 (Drosophila)) [51], *Jun *(Jun oncogene), *Adhfe1 *(alcohol dehydrogenase, iron containing, 1) [52], *Ptprr *(protein tyrosine phosphatase, receptor type, R) [53], *Klf15 *(Kruppel-like factor 15) [54,55], *Nfkb1 *(nuclear factor of kappa light polypeptide gene enhancer in B-cells 1) [26], *Sox18 *(SRY-box containing gene 18) [56,57], and *Qdpr *(quinoid dihydropteridine reductase) [58,59].

### Confirmation by quantitative RT-PCR

To confirm some of the genes that differed in expression between the iP and P.NP, quantitative RT-PCR (qRT-PCR) was performed using RNA samples of the brain regions. Ten genes were selected based on literature reports of their possible involvement in pathways related to alcohol seeking behavior (Table 2). Among the 44 comparisons with genes that significantly differed on microarrays, 35 (79%) were differentially expressed in the same direction when tested by qRT-PCR.

**Table 2:** Quantitative RT-PCR confirmation

		Ratio of expression (iP vs P.NP)^a^									
		
		Nucleus accumbens		Amygdala		Frontal cortex		Hippocampus		Caudate putamen	
						
Affymetrix ID	Gene symbol	Microarray	qRT-PCR	Microarray	qRT-PCR	Microarray	qRT-PCR	Microarray	qRT-PCR	Microarray	qRT-PCR
1368358_a_at	*Ptprr*	**2.22**	2.28	**2.47**	2.71	**2.17**	1.85	**2.42**	2.77	**1.98**	2.28
1395714_at	*Copg2 IT*	**-3.97**	-2.45	**-28.29**	-1.73	**-31.36**	-1.61	**-4.57**	-2.12	**-20.13**	-1.23
1394939_at	*Ppm1k*	**-2.05**	1.30	**-1.74**	-1.62	**-2.79**	-2.54	**-1.86**	-3.12	**-2.39**	-2.49
1379275_at	*Snx10*	**1.67**	-1.16	**2.18**	1.68	**1.94**	1.15	**1.69**	2.42	**2.02**	1.64
1380094_a_at	*Zfp212*	**1.30**	1.54	**1.22**	-1.04	**1.21**	1.19	**1.28**	1.58	**1.43**	1.86
1367734_at	*Akr1b1*	**1.22**	1.13	**1.12**	1.30	**1.27**	1.58	**1.16**	1.06	**1.25**	1.15
1379480_at	*Dgki*	**1.23**	2.72	**1.13**	-1.26	**1.17**	-2.97	**1.26**	1.11	**1.25**	-1.71
1370007_at	*Pdia4*	**1.24**	1.57	**1.34**	-1.01	[1.14]	1.05			**1.36**	-1.13
1367977_at	*Snca*					**-1.11**	-1.22	[1.07]	1.05	**-1.12**	-1.09
1387154_at	*Npy*					[-1.11]	1.01			[-1.08]	-1.20

^a^A positive number indicates the ratio of the expression level of iP/P.NP; a negative number indicates the ratio of expression level of P.NP/iP. Bold numbers in the microarray columns indicate expression is significantly different at FDR <0.05; square brackets indicate FDR between 0.05 and 0.25. qRT-PCR value is an average of six technical replicates.

### Comparison of reciprocal congenic strains

**Figure 3:**
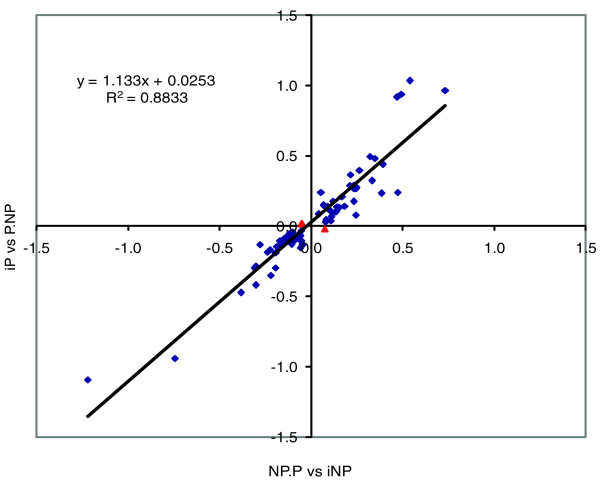
**Differential expression is highly correlated between the reciprocal congenic lines**. There were 74 probe sets within the chromosome 4 QTL that were at *P *≤ 0.05 in the same brain region (or in the average) in both experiments, and with a consistent expression direction (Table 3). Data from the average of brain regions was plotted as Log2 of the expression in NP.P/iNP (x-axis) versus log2 ratio of iP/P.NP (y-axis). Three probe sets have the same expression direction in the same brain region but not in the average of brain regions (red triangles) and include: EST-similar to Diacylglycerol kinase iota (DGKi); EST-0.79 Kb at 5' side of similar to contactin associated protein-like 2 isoform a (LOC500105); and *actin*, *gamma 2*, *smooth muscle*, *enteric *(*Acgt2*).

Previously published data comparing expression in NP.P versus iNP congenics [47] were compared to the present data (iP versus P.NP) to identify probe sets that exhibited consistent expression differences between the two experiments. For both experiments we calculated the ratio of expression from the animals carrying the iP QTL region to that from the animals carrying the iNP QTL region (that is, NP.P/iNP and iP/P.NP). Because the earlier experiment was less powerful (comparing only six animals from each strain) and because we could use the consistency of results from the two experiments to filter out false positives, we relaxed the level of significance to *P *≤ 0.05 for this comparison to reduce false negatives. Any false positives introduced by this relaxation should not be consistent between the two independent experiments. A total of 74 probesets that were significant in the two experiments (at *P *≤ 0.05) in the same brain region or in the average of the brain regions and with consistent direction in both experiments were identified (Table 3). Additional robust multi-chip average (RMA) data and uncorrected *P*-value data are included (Table S3 in Additional file 1). All of the reproducible probe sets were located within the chromosome 4 QTL interval, and therefore *cis*-regulated. The expression differences of these 74 *cis*-regulated genes were highly correlated in the two experiments (R^2 ^= 0.88; Figure 3); 71 showed expression differences of similar amounts in the same direction in both experiments. Thus, these *cis*-regulated genes are strong candidates for affecting alcohol preference. Even though the iP versus P.NP comparison identified 85 significant *trans*-regulated probe sets in at least one brain region and 206 significant probe sets when the data from all 5 regions was averaged (FDR ≤ 0.25; Table 1), no *trans*-regulated probe set was common to both experiments.

**Table 3:** Significant probe sets identified by comparison of reciprocal congenic strains

			Ratio of expression (iP vs P.NP and NP.P vs iNP)^a^											
			
			Amygdala		Nucleus accumbens		Frontal cortex		Hippocampus		Caudate putamen		Combined regions	
			
Probe set	Symbol	Gene title	iP vs. P.NP	NP.P vs. iNP	iP vs. P.NP	NP.P vs. iNP	iP vs. P.NP	NP.P vs. iNP	iP vs. P.NP	NP.P vs. iNP	iP vs. P.NP	NP.P vs. iNP	iP vs. P.NP	NP.P vs. iNP
1399134_at	LOC500054	similar to POT1-like telomere end-binding protein	-1.13	-1.11	-1.13	-1.11	**-1.07**	**-1.18**	**-1.13**	**-1.22**	-1.06	-1.07	**-1.10**	**-1.14**
1386777_at	LOC500054	similar to POT1-like telomere end-binding protein	-1.04	-1.13	-1.10	-1.35	-1.10	-1.21	**-1.19**	**-1.29**	-1.05	-1.11	**-1.10**	**-1.21**
1382865_at	Tsga14	testis specific gene A14	-1.06	-1.09	-1.13	-1.06	-1.05	-1.06	-1.11	-1.10	-1.00	-1.14	**-1.07**	**-1.09**
1382409_at	Tsga14	testis specific gene A14	-1.06	-1.12	-1.06	-1.16	-1.09	-1.02	-1.04	-1.08	-1.09	-1.01	**-1.07**	**-1.08**
1383828_at	Tsga13	EST-testis specific gene A13 (predicted)	**-1.25**	**-1.32**	**-1.45**	**-1.25**	**-1.19**	**-1.26**	**-1.57**	**-1.21**	**-1.26**	**-1.13**	**-1.34**	**-1.23**
1369895_s_at	Podxl	podocalyxin-like	1.04	-1.01	1.00	1.15	1.05	1.01	1.04	1.08	1.03	1.06	**1.03**	**1.06**
1378956_at	---	EST-similar to plexin A4	1.55	ND	2.16	ND	**1.73**	**1.55**	**1.95**	**1.39**	2.28	ND	**1.91**	**1.41**
1389291_at	Chchd3	coiled-coil-helix-coiled-coil-helix domain containing 3	**-1.09**	**-1.10**	**-1.06**	**-1.13**	**-1.06**	**-1.15**	**-1.11**	**-1.10**	**-1.05**	**-1.16**	**-1.08**	**-1.13**
1378824_at	---	EST-4.8 Kb at 3' side of similar to solute carrier family 35, member B4	1.06	1.08	1.09	ND	1.10	1.08	1.03	1.04	1.09	ND	**1.07**	**1.10**
1367734_at	Akr1b1	aldo-keto reductase family 1, member B1	**1.12**	**1.12**	**1.22**	**1.13**	**1.27**	**1.29**	**1.16**	**1.11**	**1.25**	**1.24**	**1.20**	**1.18**
1395190_at	Akr1b10	aldo-keto reductase family 1, member B10	**1.28**	**1.12**	**1.55**	**1.27**	**1.21**	**1.21**	1.34	1.05	**1.23**	**1.38**	**1.32**	**1.20**
1382034_at	Akr1b10	aldo-keto reductase family 1, member B10	1.19	-1.02	-1.41	-1.08	1.09	-1.16	-1.17	-1.05	**1.12**	**1.12**	-1.02	-1.04
1383551_at	Bpgm	2,3-bisphosphoglycerate mutase	**1.12**	**1.10**	1.14	-1.07	**1.13**	**1.16**	**1.14**	**1.15**	**1.10**	**1.10**	**1.13**	**1.09**
1388544_at	Bpgm	2,3-bisphosphoglycerate mutase	1.09	1.08	**1.10**	**1.11**	**1.11**	**1.14**	**1.10**	**1.13**	1.08	1.06	**1.10**	**1.10**
1390042_at	Tmem140	transmembrane protein 140	**1.21**	**1.32**	1.38	1.24	1.14	1.13	**1.11**	**1.14**	1.27	1.06	**1.22**	**1.18**
1383598_at	Wdr91	WD repeat domain 91 (Wdr91)	**1.33**	**1.34**	1.30	ND	**1.47**	**1.27**	**1.50**	**1.23**	**1.46**	**1.26**	**1.41**	**1.25**
1378125_at	---	EST-0.5 Kb at 3' side of similar to HSPC049 protein	**1.32**	**1.28**	**1.46**	**1.22**	**1.35**	**1.32**	**1.42**	**1.24**	**1.42**	**1.31**	**1.40**	**1.27**
1373746_at	Wdr91	WD repeat domain 91	-1.21	-1.09	-1.30	-1.14	**-1.20**	**-1.13**	**-1.26**	**-1.14**	**-1.18**	**-1.23**	**-1.23**	**-1.14**
1373190_at	Cnot4	CCR4-NOT transcription complex, subunit 4	1.00	1.09	1.02	1.16	1.02	1.01	1.07	1.14	1.03	1.00	**1.03**	**1.08**
1388441_at	LOC689574	hypothetical protein LOC689574	-1.10	-1.03	1.02	-1.06	**-1.05**	**-1.13**	-1.08	-1.10	-1.04	-1.09	**-1.05**	**-1.08**
1377890_at	---	EST-4.9 Kb at 3' side of solute carrier family 13, member 4	**1.17**	**1.50**	**1.22**	**1.34**	**1.16**	**1.30**	**1.14**	**1.23**	**1.19**	**1.19**	**1.18**	**1.31**
1392510_at	Fam180a	family with sequence similarity 180, member A	**1.22**	**1.49**	**1.78**	**1.43**	1.13	1.08	1.15	1.24	1.08	1.11	**1.25**	**1.26**
1391721_at	---	EST-2.5 Kb at 5' side of cholinergic receptor, muscarinic 2	-1.55	ND	-2.91	ND	**-1.82**	**-2.10**	**-1.71**	**-1.63**	-1.88	ND	**-1.92**	**-1.67**
1379480_at	Dgki	diacylglycerol kinase, iota	**1.13**	**1.14**	**1.23**	**1.22**	**1.17**	**1.24**	1.26	1.09	**1.25**	**1.27**	**1.21**	**1.19**
1395107_at	Dgki	EST-similar to diacylglycerol kinase iota	-1.02	1.04	-1.15	1.06	1.01	-1.01	**1.10**	**1.16**	-1.01	1.02	-1.01	1.05
1393410_at	---	EST-0.79 Kb at 5' side of similar to contactin associated protein-like 2 isoform a	1.00	-1.18	1.09	1.15	**-1.09**	**-1.11**	1.02	-1.06	1.03	1.00	1.01	-1.04
1390393_at	---	EST-5 Kb at 5' side of similar to contactin associated protein-like 2 isoform a	**-1.08**	**-1.15**	-1.01	1.01	**-1.16**	**-1.21**	-1.03	-1.12	-1.03	-1.07	**-1.06**	**-1.11**
1370007_at	Pdia4	protein disulfide isomerase associated 4	**1.34**	**1.24**	1.24	1.10	**1.14**	**1.19**	1.06	1.12	**1.36**	**1.14**	**1.22**	**1.16**
1397447_at	Zfp398	zinc finger protein 398	-1.04	-1.13	-1.08	-1.08	-1.04	1.01	-1.04	-1.02	-1.06	1.01	**-1.05**	**-1.04**
1380094_a_at	Zfp212	zinc finger protein 212	1.22	ND	1.30	ND	1.21	ND	**1.28**	**1.15**	1.43	ND	**1.29**	**1.16**
1390625_at	RGD1304879	similar to zinc finger protein 398 (zinc finger DNA binding protein p52/p71)	**1.43**	**1.40**	**1.27**	**1.39**	**1.33**	**1.20**	**1.30**	**1.36**	**1.46**	**1.22**	**1.36**	**1.31**
1377600_at	Znf777	zinc finger protein 777	1.08	1.10	1.07	-1.00	**1.09**	**1.12**	1.13	1.08	1.03	1.10	**1.08**	**1.08**
1375914_at	Krba1	KRAB-A domain containing 1	-1.04	-1.07	-1.07	1.04	-1.07	-1.02	-1.05	-1.14	**-1.06**	**-1.12**	**-1.06**	**-1.06**
1371691_at	Rarres2	retinoic acid receptor responder (tazarotene induced) 2	-1.14	-1.01	1.12	-1.09	**-1.16**	**-1.22**	-1.23	-1.19	-1.01	-1.08	**-1.08**	**-1.11**
1376401_at	RGD1561107	EST-replication initiator 1	1.12	1.10	**1.16**	**1.13**	**1.19**	**1.12**	**1.13**	**1.13**	**1.18**	**1.14**	**1.15**	**1.12**
1382755_at	Tra2a	rranscribed locus	1.11	1.16	-1.13	-1.12	1.07	1.20	**1.13**	**1.32**	1.11	1.43	**1.06**	**1.19**
1387154_at	Npy	neuropeptide Y	-1.04	-1.19	-1.06	1.12	**-1.11**	**-1.11**	-1.09	-1.14	-1.08	-1.05	**-1.08**	**-1.07**
1380062_at	Mpp6	membrane protein, palmitoylated 6 (MAGUK p55 subfamily member 6)	1.02	1.00	1.03	-1.09	1.07	1.04	**1.13**	**1.19**	1.06	1.02	1.06	1.03
1383324_at	Mpp6	membrane protein, palmitoylated 6 (MAGUK p55 subfamily member 6)	1.01	1.09	1.10	-1.01	**1.11**	**1.12**	**1.10**	**1.20**	1.06	1.09	**1.07**	**1.10**
1397419_at	Mpp6	membrane protein, palmitoylated 6 (MAGUK p55 subfamily member 6)	-1.02	1.12	1.12	1.01	**1.18**	**1.13**	**1.14**	**1.22**	1.07	1.10	**1.09**	**1.11**
1397949_at	---	EST-similar to MAGUK p55 subfamily member 6	-1.00	1.13	1.16	1.06	**1.15**	**1.18**	**1.15**	**1.20**	1.07	1.12	**1.10**	**1.14**
1398627_at	---	EST- similar to MAGUK p55 subfamily member 6	-1.01	1.04	1.05	1.10	**1.09**	**1.09**	**1.07**	**1.16**	1.04	1.02	**1.05**	**1.08**
1384136_at	Osbpl3	oxysterol binding protein-like 3	-1.06	-1.03	-1.09	1.07	-1.15	-1.03	**-1.12**	**-1.16**	-1.15	-1.06	-1.11	-1.04
1378543_at	Hnrnpa2b1	EST-heterogeneous nuclear ribonucleoprotein A2/B1 (predicted)	**-1.31**	**-1.23**	-1.26	-1.18	**-1.17**	**-1.35**	**-1.16**	**-1.16**	**-1.19**	**-1.25**	**-1.22**	**-1.23**
1371395_at	Cbx3	chromobox homolog 3 (HP1 gamma homolog, Drosophila)	-1.07	-1.07	-1.04	-1.00	-1.04	-1.02	-1.03	-1.03	**-1.05**	**-1.10**	**-1.04**	**-1.04**
1379275_at	Snx10	sorting nexin 10	**2.18**	**1.40**	1.67	-1.05	**1.94**	**1.58**	**1.69**	**1.58**	**2.02**	**1.55**	**1.89**	**1.39**
1383585_s_at	Snx10	EST-sorting nexin 10	**-1.10**	**-1.17**	-1.08	-1.05	-1.12	-1.09	-1.06	-1.03	-1.08	-1.26	**-1.09**	**-1.12**
1377198_at	---	EST-2 Kb at 3' side of src family associated phosphoprotein 2	**-1.23**	**-1.33**	-1.16	-1.09	**-1.10**	**-1.09**	**-1.10**	**-1.19**	-1.03	-1.15	**-1.12**	**-1.17**
1369979_at	Skap2	src family associated phosphoprotein 2	**-1.20**	**-1.22**	-1.11	-1.04	-1.03	-1.16	-1.05	-1.07	1.01	-1.12	**-1.07**	**-1.12**
1388118_at	Hibadh	3-hydroxyisobutyrate dehydrogenase	-1.07	-1.01	-1.01	-1.04	**-1.05**	**-1.09**	-1.05	-1.03	-1.06	-1.05	**-1.05**	**-1.04**
1378742_at	LOC682099	EST-similar to juxtaposed with another zinc finger protein 1	**2.05**	**1.64**	**1.92**	**1.80**	**2.11**	**1.71**	**1.85**	**1.76**	**1.83**	**1.43**	**1.95**	**1.66**
1379629_at	---	EST-4.7 kb at 5' side of similar to cAMP responsive element binding protein 5 isoform alpha	**-1.38**	**-1.35**	**-1.40**	**-1.37**	**-1.34**	**-1.27**	**-1.42**	**-1.20**	**-1.41**	**-1.34**	**-1.39**	**-1.30**
1394833_at	---	EST-0.6 Kb at 5' side of beta chimerin	-1.12	-1.15	-1.04	-1.19	-1.08	1.02	-1.06	-1.10	1.08	-1.03	**-1.04**	**-1.09**
1370648_a_at	Wipf3	WAS/WASL interacting protein family, member 3	1.01	1.18	-1.01	1.00	-1.01	1.09	1.00	-1.10	**1.11**	**1.12**	1.02	1.06
1392541_at	Ggct	gamma-glutamyl cyclotransferase	**-1.34**	**-1.19**	-1.28	-1.06	**-1.26**	**-1.26**	-1.18	-1.08	**-1.33**	**-1.26**	**-1.28**	**-1.16**
1398107_at	Ggct	gamma-glutamyl cyclotransferase	**-1.10**	**-1.15**	-1.02	1.14	-1.17	ND	-1.06	-1.07	-1.15	-1.00	-1.10	-1.03
1394973_at	Pde1c	EST-cyclic nucleotide phosphodiesterase 1 C	1.14	-1.01	1.08	1.09	1.02	1.02	-1.02	-1.01	**1.37**	**1.16**	1.11	1.05
1375640_at	Fkbp9	FK506 binding protein 9	-1.05	1.28	1.23	1.01	1.15	1.04	1.02	1.01	1.07	1.05	**1.08**	**1.07**
1388493_at	Ecop	EGFR-coamplified and overexpressed protein	-1.05	-1.10	-1.04	-1.00	**-1.10**	**-1.09**	-1.05	-1.05	**-1.11**	**-1.09**	**-1.07**	**-1.06**
1396215_at	---	EST-similar to RIKEN cDNA 2610022G08	1.01	-1.07	-1.07	-1.10	-1.03	-1.07	-1.08	-1.07	**-1.14**	**-1.20**	**-1.06**	**-1.10**
1394939_at	Ppm1k	protein phosphatase 1 K (PP2C domain containing)	**-1.74**	**-2.67**	**-2.05**	**-2.36**	**-2.79**	**-2.57**	**-1.86**	**-2.05**	**-2.39**	**-2.05**	**-2.13**	**-2.33**
1392921_at	Ppm1k	Protein phosphatase 1 K (PP2C domain containing)	**-1.07**	**-1.22**	**-1.21**	**-1.19**	**-1.12**	**-1.16**	**-1.14**	**-1.22**	**-1.15**	**-1.12**	**-1.14**	**-1.18**
1388778_at	---	EST-3.6 Kb at 5' side of similar to protein phosphatase 1 K (PP2C domain containing)	**-1.27**	**-1.27**	-1.27	-1.17	**-1.18**	**-1.27**	**-1.22**	**-1.23**	**-1.22**	**-1.26**	**-1.23**	**-1.24**
1367977_at	Snca	synuclein, alpha	1.03	-1.08	-1.04	-1.11	**-1.10**	**-1.09**	1.07	1.03	**-1.12**	**-1.12**	**-1.03**	**-1.07**
1385271_at	RGD1565731	EST-similar to KIAA1680 protein (predicted)	-1.02	1.04	-1.05	-1.08	-1.03	-1.02	**-1.20**	**-1.11**	-1.09	-1.01	-1.08	-1.04
1391945_at	---	Transcribed locus	**2.01**	**1.33**	**2.37**	**1.60**	**1.54**	**1.38**	**1.88**	**1.30**	**2.61**	**1.70**	**2.05**	**1.45**
1393607_at	Grid2	EST-glutamate receptor, ionotropic, delta 2	**-1.27**	**-1.34**	-1.13	1.04	**-1.17**	**-1.14**	-1.12	-1.23	-1.02	-1.08	**-1.14**	**-1.14**
1386869_at	Actg2	actin, gamma 2, smooth muscle, enteric	1.03	1.05	1.07	-1.11	1.03	-1.00	**-1.06**	**-1.10**	-1.01	-1.02	1.01	-1.03
1379610_at	---	EST	**1.19**	**1.31**	-1.00	ND	1.14	1.03	1.24	1.07	-1.03	ND	1.10	1.06
1376481_at	Adamts9	a disintegrin-like and metalloprotease (reprolysin type) with thrombospondin type 1 motif, 9	1.09	1.30	1.16	ND	1.22	ND	**1.30**	**1.28**	1.27	ND	**1.20**	**1.18**
1376747_at	---	EST, strongly similar to membrane associated guanylate kinase, WW and PDZ domain containing 1 isoform b [Mus musculus]	-1.11	-1.22	1.00	1.05	**-1.25**	**-1.12**	1.06	1.02	**-1.20**	**-1.13**	**-1.09**	**-1.08**
1381871_at	NA	Transcribed locus	1.21	1.20	-1.05	1.90	**1.28**	**1.49**	**1.31**	**1.42**	1.19	1.08	**1.18**	**1.39**
1384504_at	Magi1	membrane associated guanylate kinase, WW and PDZ domain containing 1	1.15	1.05	1.05	1.41	**1.16**	**1.20**	1.20	1.08	1.08	1.17	**1.13**	**1.17**
1397438_at	Magi1	membrane associated guanylate kinase, WW and PDZ domain containing 1	1.26	1.01	1.12	1.09	ND	1.02	1.26	ND	**1.17**	**1.08**	1.18	1.04

Comparison of iP versus P.NP (this paper) and NP.P versus iNP [47] data. Probe sets that were significant (at *P *≤ 0.05) with consistent direction in at least one brain region or in the average of the brain regions were analyzed. ^a^Positive number is the ratio of the expression level of iP/P.NP (this paper) or NP.P/iNP [47] (that is, in both cases expression is higher in the strain with the P alleles in the introgressed region); negative numbers indicate the ratio of expression level of P.NP/iP (this paper) or iNP/NP.P [47]. Bold numbers indicate significant ratio of expression. ND indicates not detectable. The probe sets were sorted by genomic location; all are on chromosome 4.

## Discussion

In this study, the iP background strain was compared to the P.NP congenic strain, which has the iNP chromosome 4 QTL interval between markers *D4Rat119 *and *D4Rat55 *introgressed onto the iP background. Because the congenic and background strains are identical except for the region on chromosome 4, the *a priori *expectation is that only *cis*-regulated genes located in that region of chromosome 4 or genes *trans*-regulated by genes within that region should differ. This is expected to be a small set of genes, the signal from which could be masked by random variations in the very large set of genes that do not differ. Among *cis*-regulated differentially expressed probe sets, only 53 out of 157 were significant in a single brain region. Among the other 104 probe sets, 102 differed in the same direction in at least two regions. Many genes are expected to be expressed under similar regulatory control in different brain regions, so we also conducted an analysis of the average expression levels across the five regions and identified additional genes. The magnitude of the differences was small. Other comparisons of gene expression in rat brain have also reported small differences [47,58,60-62].

These findings from the iP versus P.NP congenic strain were then compared with previous transcriptome profiling of the reciprocal NP.P congenic strain versus iNP background strain [47]. We identified 74 *cis*-regulated probe sets with consistent direction and magnitude of expression differences in the two experiments (Figure 3; Table 3). These are strong candidates for influencing the alcohol preference phenotype. The differences in gene expression, although small, were quite consistent between experiments for these *cis*-regulated genes (Table 3, Figure 3). This is noteworthy since the experiments were completely independent, done at two different times using different strains (NP.P versus iNP and iP versus P.NP) bred at different times, and demonstrates the reproducibility of transcriptome profiling on microarrays.

In these comparisons between congenic animals, the only genes outside the chromosome 4 QTL region that are expected to show differential expression are those that are *trans*-regulated by genes lying within the region. Fewer *trans*-regulated genes showed differential expression in any one brain region, whereas analyzing the average expression values resulted in more *trans*-regulated genes (Table 1). However, most of these were not common to the reciprocal congenic experiment [47], suggesting that most of these *trans*-differences could be false positives.

Of the 74 *cis*-regulated candidate genes common to the reciprocal congenic experiments and the most significant *trans*-regulated candidate genes from the iP vs P.NP comparison, 10 genes were chosen for PCR confirmation based on their expression differences and/or literature reports of their possible involvement in pathways related to alcohol-seeking behavior. Of these, 79% showed consistent direction of expression, in part because RT-PCR is a logarithmic process and not as good for detecting small differences in expression (Table 2). The primers for these confirmation studies, when possible, were in the coding sequences spanning an intron. It has been our experience that when primers are designed based on the coding regions, as we did here, the number of confirmed genes is lower (50 to 70%) than when using primers designed within the 3' sequences used on the microarray chips (80 to 90%), perhaps due in part to alternative splicing or 3' untranslated regions. A limitation of this confirmation was that samples were pooled by brain region, limiting the statistical power for data analysis.

Sorting nexin10 (*Snx10*) is one of the most significant genes identified in both reciprocal congenics. Snx10 protein is a member of sorting nexins, a diverse group of cellular trafficking proteins that are unified by the presence of a phospholipid-binding motif, the PX domain. Snx10 protein may be involved in the regulation of endosome homeostasis [63]. In four of the brain regions we studied, the animals with the iP chromosome 4 QTL segment (iP and NP.P) demonstrated a higher expression of *Snx10 *mRNA than those with the iNP segment (iNP and P.NP; Table 3).

Ppm1k is a serine/threonine protein phosphatase. Together with other protein kinases, these enzymes control the state of phosphorylation of cell proteins and thereby provide an important mechanism for regulating cellular activity.

**Figure 4:**
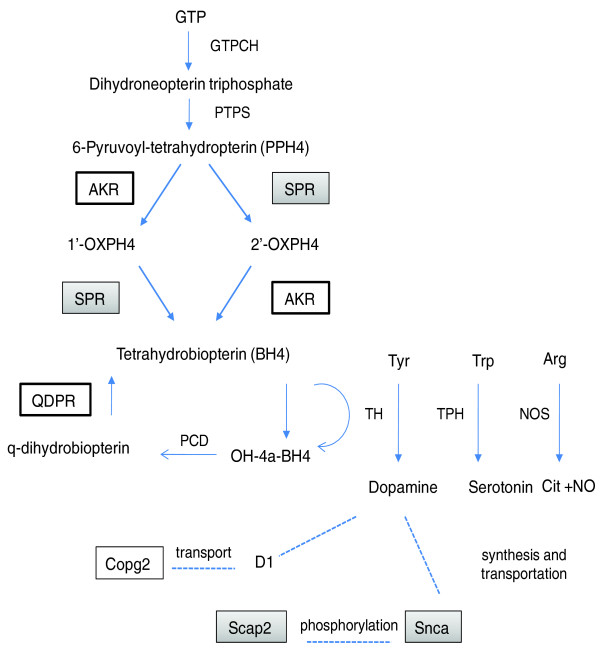
**Candidate genes in the dopamine and serotonin system**. Sepiaperterin reductase (SPR) and aldo-keto reductase (AKR) reduces an intermediate, 6-pyruvoyl-tetrahydropterin (PPH4), to 1'-OXPH4, or 2'-OXPH4, and catalyzes the final step of tetrahydrobiopterin (BH4) synthesis, an essential cofactor for phenylalanine hydroxylase, tyrosine hydroxylase (TH), tryptophan hydroxylase (TPH) and nitric oxide synthase (NOS) [65,66]. Quinoid dihydropteridine reductase (QDPR) mediates reduction of quinonoid dihydrobiopterin. Several candidate genes are related to dopamine function. Snca regulates dopamine biosynthesis and attenuates dopamine transporter activity. Scap2 phosphorylates Snca, and Copg2 is involved in the transport of the dopamine receptor 1 (D1). Arrows represent metabolic steps, and dashed lines represent genes that are functionally related. Identified candidate genes are in boxes; gray color indicates a lower expression in iP and white color indicates higher expression in iP. GTPCH, GTP-cyclohydrolase I; PTPS, 6-pyruvoyltetrahydropterin synthase; 1'-OXPH4, 1'-oxo-2'-hydroxypropyl tetrahydropterin; 2'-OXPH4, 1'-hydroxy-2'-oxo-tetrahydropterin; OH-4a-BH4, pterin-4a-carbinolamine; PCD, pterin-4a-carbinolamine dehydratase.

Aldo-keto reductase 1 member B1 (Akr1b1), and Akr1b10 catalyze the reduction of aliphatic and aromatic aldehydes to their corresponding alcohols. These two genes are both expressed at higher levels in the animal with the P chromosome 4 interval than the animal with the iNP chromosome 4 interval in both iP versus P.NP and NP.P versus iNP comparisons. Although sepiaperterin reductase (SPR) is known to be the major enzyme in the tetrahydrobiopterin (BH4) synthesis, aldo-keto reductases (AKRs) and carbonyl reductases (CBRs) can also convert 6-pyruvoyltetrahydropterin to BH4 [64-66], which is an essential cofactor for tyrosine hydroxylase (TH) and tryptophan hydroxylase (TPH), both of which are involved in dopamine and serotonin biosynthesis (Figure 4). Alcohol is known to interact with the dopamine and serotonin neurotransmitter systems in the brain.

Diacylglycerol kinase (Dgki) regulates the levels of various pools of diacylglycerol (DAG), affecting DAG-mediated signal transduction. We found that *Dgki *mRNA is expressed at higher levels in animals with the iP chromosome 4 QTL interval (iP and NP.P) than those with the iNP interval (P.NP and iNP) in all the brain regions studied. *Dgki *mRNA has been shown to be expressed at higher levels in discrete brain regions of the alcohol accepting (AA) rats than in the alcohol non-accepting (ANA) rats [67]. The highest mRNA expression of *Dgki *was found in the human brain [68]. Dgki is expressed in the cytoplasm of most dorsal root ganglion neurons, through which primary afferent information passes *en route *to the brain [69]. Dgki catalyzes the phosphorylation of DAG, an activator of protein kinase C, to phosphatidic acid, and thus down-regulates second messenger pathways activated by protein kinase C, which play important roles in regulating behavioral responses to ethanol [70].

Protein disulfide isomerase family A, member 4 (Pdia4), also known as endoplasmic reticulum p72 (ERp72) [71], functions in disulfide bond formation and isomerization. Together with other endoplasmic reticulum-resident molecular chaperones, Pdia4 protein participates in critical steps in the folding of apolipoprotein B before any substantial lipidation occurs. *Pdia4 *mRNA was differentially expressed in four microarray gene profiling studies using animals selected for high and low alcohol consumption, which include iP versus iNP [58], inbred high-alcohol-drinking (iHAD) versus inbred low-alcohol-drinking (iLAD) (unpublished data), NP.P versus iNP [47], and iP versus P.NP (this paper). In all these studies, the animals with the high drinking allele had higher levels of *Pdia4 *mRNA than the animals that had the low drinking allele.

NPY is one of the most abundant neuropeptides in the central nervous system, and has been shown to have multiple functions, including regulation of feeding behavior, anxiety, addiction, bone density and memory retention [72,73]. In the present study, *Npy *expression has the same trend in all five brain regions, with lower expression in animals with the iP chromosome 4 QTL interval; this is consistent with previous findings of lower expression in iP than in iNP animals [43]. Alcohol consumption is inversely related to NPY levels in the brain [43,74]. Intracerebroventricular administration of NPY significantly decreased ethanol intake in P rats [75].

*Snca *is a previously identified candidate gene for alcohol consumption in the iP/iNP animals [42,47], and has been associated with craving and alcohol dependence in humans [31,76]. In both microarray comparisons,*Snca *was found expressed at lower levels in the frontal cortex and caudate of animals with the iP QTL interval. However, an opposite trend was observed in the hippocampus, where *Snca *was previously shown to have higher expression in iP rats [42]. Higher mRNA and protein levels have been observed in serum from alcoholic patients compared to that from controls [77,78]. *SNCA *has been associated with craving and alcohol dependence in humans [31,76]. *Skap2 *and *Fyn*-kinase were previously identified as being involved in the phosphorylation of *Snca *(Figure 4). *Scap2 *is expressed at lower levels in NP.P than iNP and also lower in iP than P.NP; it inhibits the phosphorylation of *Snca *and acts as a substrate for the Src family of kinases, such as Fyn [79]. Fyn specifically phosphorylates tyrosine residue 125 of Snca [80]. Snca and Fyn are co-localized in subcellular structures and expressed in similar brain regions [80]. Miyakawa and colleagues found that Fyn-kinase is involved in ethanol sensitivity through NMDA-receptor function [81]. Thus, these genes could work in concert to control alcohol seeking behavior.

A limitation of microarray technology is that a SNP that differs between the two strains tested could affect the hybridization to a probe set in a way that mimics an expression difference. Because expression data are composites from many probe sets, this is likely to make only a small difference. To address this possibility, individual probes within each of the 74 strong candidate probe sets were analyzed. There were no detectable SNP effects in 71 of these genes; only 3 genes had one probe that differed from the overall pattern (data not shown). This indicated that the majority of expression differences detected in this study were not the result of SNP effects.

Ingenuity Pathways Analysis (Ingenuity Systems, Inc., Mountain View, CA, USA) of the genes significant in either experiment (iP versus P. NP or NP.P versus iNP, at FDR <0.25) was performed. The dopamine and serotonin biosynthesis and other pathways - for example, the Nfkb1 pathway - were overrepresented. Six candidate genes, including *Akr1b1*, *Qdpr*, *Snca*, *Spr*, *Scap2*, and *Copg2*, are directly or indirectly involved with the dopamine and serotonin biosynthesis pathway (Figure 4). Confirmation of candidate genes in the Nfkb1 pathway, which is associated with alcohol dependence [26], is ongoing.

## Conclusions

Two independent gene profiling experiments using reciprocal congenic strains have identified strong, *cis*-acting candidate genes for alcohol consumption within the chromosome 4 QTL region. These findings provide important candidate genes for future functional and knockout studies.

## Materials and methods

### Animals

Creation of the P.NP-(*D4Rat119 *(62.8 Mb)-*D4Rat55 (*127.9 Mb) congenic strain has been previously described [44]. Briefly, it was initiated by crossing one male rat from the iNP strain with one female rat from the iP strain to create iP × iNP F1 animals, which were backcrossed to the iP strain to produce the N2 generation. Ten generations of backcrossing to the iP strain were performed, followed by an intercross between N10 animals to produce homozygous animals (N10F1), which resulted in the finished congenic P.NP strain (Figure 1).

Presence of the chromosome 4 interval was confirmed using four to five microsatellite markers, including *D4Rat119 *and *D4Rat55*. Microsatelitte markers at 47.8 Mb (*D4Rat15*) and 159.3 Mb (*D4Rat192*) defined the extent of the introgressed region for both the P.NP and the NP.P congenic strains. At microsatelitte markers 62.8 Mb (*D4Rat119*) and 127.9 Mb (*D4Rat55*), the NP.P strain was homozygous for the iP allele and the P.NP strain was homozygous for the iNP allele. Although the locations of the recombination boundaries have not been resolved, they are between 62.8 Mb and 47.8 Mb and between 127.9 Mb and 159.3 Mb [44]. The QTL map in Figure 2 was generated using our published data [51] plus additional markers using MAPMAKER/EXP82; the 95% confidence interval was calculated [83] and it spans 54.8 Mb to 105 Mb.

All animal housing and handing was as previously described [47]. The animals used in these experiments were maintained in facilities fully accredited by the Association for the Assessment and Accreditation of Laboratory Animal Care (AAALAC). All research protocols were approved by the Institutional Animal Care and Use Committee and are in accordance with the guidelines of the Institutional Animal Care and Use Committee of the National Institute on Drug Abuse, NIH, and the *Guide for the Care and Use of Laboratory Animals *(Institute of Laboratory Animal Resources, Commission on Life Sciences, National Research Council 1996).

A total of 16 (8 iP and 8 P.NP) male rats, 14 to 15 weeks of age, were sacrificed by decapitation between 0900 and 1000 hours over two consecutive days, with equal numbers of animals from each strain sacrificed each day. The head was immediately immersed in chilled isopentane (-50°C) for 15 seconds and then placed in a cold box maintained at -15°C, where the brain was rapidly removed and placed on a glass plate for dissection. All equipment used to obtain tissue was treated with RNaseZap (Ambion, Inc. Austin, TX, USA) to prevent RNA degradation. The amygdala, nucleus accumbens, caudate putamen, frontal cortex, and hippocampus were dissected as previously described [84].

### RNA isolation

Dissected tissues were immediately homogenized in Trizol reagent (Invitrogen, Carlsbad, CA, USA) and processed according to the manufacturer's protocol, but with triple the suggested ratio of Trizol to tissue [60]. RNA was further purified through RNeasy^® ^mini columns (Qiagen, Valencia, CA, USA), according to the manufacturer's protocol. To avoid genomic DNA contamination in the real-time PCR assay, the RNA was treated with DNase I. Total RNA yields from the iP and P.NP groups were similar (*P *> 0.4). The quality of the RNA from all rats and regions was similar, as monitored by absorbance spectra from 210 to 350 nm, by electrophoresis on 1% agarose gels, and using the Agilent Bioanalyzer to confirm the ribosomal bands.

### RNA labeling and microarray hybridization

RNA from each brain region of each individual rat was labeled and hybridized separately on an Affymetrix Rat Genome 230 2.0 microarray. Starting with 5 μg of total RNA from each animal, biotinylated cRNA was produced using the GeneChip^® ^Expression 3' Amplification One-Cycle Target Labeling and Control Reagents kit according to Affymetrix standard protocol. Fragmented, biotinylated cRNA (15 μg) was mixed into 300 μl of hybridization cocktail, of which 200 μl was used for each hybridization. Hybridization was for 17 hours at 42°C. Washing, staining, and scanning were carried out according to the standard protocol.

To minimize systematic errors, all stages of the experiment were balanced across phenotypes. That is, equal numbers of P.NP and iP animals were sacrificed each day, and equal numbers of RNA preparations from iP and P.NP animals were processed through the labeling, hybridization, washing and scanning protocols on each day, in different alternating orders. Whenever possible, common premixes of reagents were used.

### Data analysis and informatics

Each GeneChip^® ^was scanned using an Affymetrix Model 3000 scanner and underwent image analysis using Affymetrix GCOS software. Microarray data are available from the National Center for Biotechnology Information's Gene Expression Omnibus [85,86], under series accession [GEO:GSE15415] [87]. Raw cel files were imported into the statistical programming environment R for further analysis with tools available from the Bioconductor Project [88]. Expression data were normalized and log_2 _transformed using the RMA method [89,90] implemented in the Bioconductor package RMA.

Our primary hypothesis was that *cis*-regulated genes within the QTL were responsible for the strain differences; thus, to detect genes within the region that differed between the P.NP and iP rats, the probe sets that mapped to the chromosome 4 QTL region between microsatellite markers *D4Rat151 *and *D4Rat55 *that flanked the introgressed region (from 29,413,686 to 128,186,835 bases) were analyzed using *t*-tests, calculated using the package Limma [91]. To increase power and decrease the false discovery rate [92], probe sets not reliably detected on at least one-third of the microarrays in at least one experimental group (using the Affymetrix Microarray Analysis Suite 5.0 detection call) were not analyzed [93]. For the analyses of a specific brain region, the QTL probe sets were retained if present on at least one-third of the microarrays for either the congenic P.NP or iP animals (number of probe sets detected ranged from 644 to 694). To detect differences in gene expression common to several regions, data from the five discrete brain regions of each animal were averaged. This reduces random technical variation from the individual extractions and labeling, and thereby provides more power to detect differences that are in the same direction in multiple regions but may fall below significance in individual regions. For the analyses of average expression level, QTL probe sets were retained if present on at least one-third of the microarrays in at least one brain region in at least one strain (690 probe sets).

Secondary analyses examined expression differences elsewhere in the genome that could arise from *trans*-acting factors within the region. For the analyses of a specific brain region, the probe sets were retained if present on at least one-third of the microarrays for either the congenic P.NP or iP animals (21,345 to 22,994 probe sets). For the analyses of average expression level, probe sets were retained if present on at least one-third of the microarrays in at least one brain region in at least one strain (23,050 probe sets).

### Comparison of reciprocal congenics

Previously published data comparing expression in NP.P versus iNP congenics [47] were compared to the present data (iP versus P.NP) to identify probe sets that exhibited consistent expression differences between the two experiments. For both experiments we calculated the ratio of expression from the animals carrying the iP QTL region to that from the animals carrying the iNP QTL region (that is, NP.P/iNP and iP/P.NP). Thus, for both experiments, a positive ratio of expression represents higher expression in the animals with the iP chromosome 4 QTL interval (iP and NP.P), and a negative value represents lower expression in the animals with iP chromosome.

Because the earlier experiment was less powerful (comparing only six animals from each strain) and because we could use the consistency of results from the two experiments to filter out false positives, we relaxed the level of significance to *P *≤ 0.05 for this comparison to reduce false negatives. Any false positives introduced by this relaxation should not be consistent between the two independent experiments. Thus, genes that were significant in the two experiments (at *P *≤ 0.05) in the same brain region or in the average of the brain regions and with consistent direction in both experiments were identified (Table 3).

### SNP effect analysis

Potential chromosomal regions containing SNPs were identified using probe, as opposed to probe set, level analysis according to the method of Rostoks, Borevitz, *et al*. [94]. Briefly, probe level expression was extracted from individual CEL files from all five brain regions after background correction. Expression levels for individual probes were averaged within animal, across brain regions, in a manner identical to that applied to probe sets. An algorithm was applied to the probes belonging to each probe set such that overall probe set group differential expression was ascertained and then each probe's expression was corrected for this. This made it easier to identify individual probes with relatively small deviations from large overall group differential expressions. For each probe set, the differential expression of each probe was then plotted using the matplot function of Bioconductor package affyPLM [95,96].

### Mapping of ESTs

In order to map the genomic location of significant ESTs, sequences were obtained from the Affymetrix website [97] and aligned to the rat genome using BLAST at NCBI [98]. Probe sets that aligned within a gene were referred to by that gene name. Probe sets that aligned between genes were listed as the nearest gene with the distance noted. ESTs that aligned to multiple loci or could not be positioned on the genome were labeled as EST.

### Quantitative real-time PCR

Ten genes were selected for confirmation in the five brain regions used in the microarray analysis, using qRT-PCR. Amplification primers were designed from the sequence in the coding region of the gene using Vector NTI (Invitrogen); when possible, at least one primer spanned an exon/intron boundary. qRT-PCR was carried out using SYBR Green chemistry and the ABI Prism 7300 Sequence Detection System (Applied Biosystems, Foster City, CA, USA) as previously described [47]. To correct for sample-to-sample variation, an endogenous control (glyceraldehyde 3-phosphate dehydrogenase, GAPDH) was amplified with the target and served as an internal reference to normalize the data. The average GAPDH Ct values for iP and P.NP were the same in each brain region tested, making this an appropriate control gene to normalize the expression of the candidate genes of interest. Relative quantification was performed using the standard curve method (Applied Biosystems, User Bulletin #2) [99]. For each pooled iP and P.NP sample, eight animals were pooled by each of five brain regions and six technical replicates were performed.

### Ingenuity pathway analysis

The interactions between differentially expressed genes in either comparisons (with FDR <0.25) were investigated using Ingenuity Pathway Analysis (IPA 5.0; Ingenuity Systems, Inc., Mountain View, CA). The differentially expressed genes were uploaded into IPA. Each gene identifier was mapped to its corresponding gene in the Ingenuity Pathway Knowledge Base, a manually curated database of interactions from literature [100]. These genes were overlaid onto a global network developed from the information contained in the Ingenuity Pathway Knowledge Base. Networks of these genes, defined as the reflection of all interactions of a given gene defined in the literature, were then algorithmically generated based on their connectivity. The interactions indicate physical association, induction/activation or repression/inactivation of one gene product by the other, directly or through another intermediary molecule.

## Abbreviations

DAG: diacylglycerol; Dgki: diacylglycerol kinase; EST: expressed sequence tag; FDR: false discovery rate; iNP: inbred alcohol-nonpreferring; iP: inbred alcohol-preferring; Mb: million bases; NP: alcohol-nonpreferring; NP.P: congenic rat in which the iP chromosome 4 QTL interval was transferred to the iNP; P: alcohol-preferring; P.NP: congenic rat in which the iNP chromosome 4 QTL interval was transferred to the iP; qRT-PCR: quantitative real-time PCR; QTL: quantitative trait locus; RMA: robust multi-chip average; SNP: single nucleotide polymorphism.

## Competing interests

The authors declare that they have no competing interests.

## Authors' contributions

TL, LC and KM developed the reciprocal congenic rats, HE and JM were responsible for microarray experiments, MK and JM performed data analysis with participation from HE, and AC performed qRT-PCR. TL, LC, HE, JM, and MK wrote the manuscript.

## Supplementary Material

Additional file 1 Supplemental data of expression profiling in alcoholpreferring and non-preferring reciprocal congenic rats. Click here for file
